# Status and influencing factors of pre-hospital delay in young and middle-aged Chinese patients with acute ischemic stroke

**DOI:** 10.3389/fpubh.2025.1539219

**Published:** 2025-06-04

**Authors:** Dan Liu, Yuqi Fang, Zhuan Yin, Qingxiang Zhu, Chaofeng Li

**Affiliations:** ^1^Hunan Provincial Key Laboratoryof Regional Hereditary Birth Defects Prevention and Control, Changsha Hospital for Maternal & Child Health Care Affiliated to Hunan Normal University, Changsha, China; ^2^Department of Emergency Medicine, Xiangya Hospital of Central South University, Changsha, China; ^3^School of Medicine of Hunan Normal University, Changsha, China

**Keywords:** young and middle-aged, acute ischemic stroke, pre-hospital delay, health literacy, social support, coping styles

## Abstract

**Objective:**

The purpose of this study was to investigate the status quo of prehospital delay in young and middle-aged AIS patients and analyze the main factors affecting prehospital delay in young and middle-aged AIS patients. Thus, it can provide reference for reducing prehospital delay in young and middle-aged AIS patients and promote secondary prevention of stroke.

**Methods:**

This cross-sectional study was conducted from May 2023 to February 2024 using convenient sampling method, and included middle-aged and young ischemic stroke patients from two top three hospitals in Hunan Province. Through questionnaire survey, social support scale, health literacy scale and medical coping style scale were used to investigate.

**Results:**

A total of 671 patients were investigated. According to the standard of 3 h from the onset of symptoms to the hospital, middle-aged and young stroke patients were divided into delayed group and non-delayed group. The results showed that 534 patients with pre-hospital delay occurred, with an incidence of 79.6%. Binary logistic regression analysis showed that the factors that extended the pre-hospital time of patients were: The onset place was at home (OR = 4.997, *p* = 0.011), the first symptoms were numbness and weakness of limbs (OR = 3.500, *p* = 0.027), avoidance coping style (OR = 1.298, *p* = 0.027) and submission coping style (OR = 1.721, *p* < 0.001). The factors that shorten the pre-hospital time of patients are: AIS was known at the time of onset (OR = 0.120, *p* = 0.001), the first symptoms were headache and vomiting (OR = 0.052, *p* < 0.001), disturbance of consciousness (OR = 0.087, *p* < 0.001), social support level (OR = 0.891, *p* = 0.001) and coping style (OR = 0.688, *p* < 0.001).

**Conclusion:**

The incidence of pre-hospital delay in young and middle-aged ischemic stroke patients is high, and the current situation is not optimistic. Factors affecting pre-hospital delay in young and middle-aged AIS patients included location of onset, first symptoms, whether they knew that the onset was AIS, level of social support, health literacy, and coping style. In the future, these aspects can be considered to reduce the delayed development of AIS in young and middle-aged people before hospital and promote secondary prevention of stroke.

## 1 Introduction

Cerebral Stroke is a common cerebrovascular disease. It refers to a group of diseases that cause brain damage due to blocked or broken blood vessels so that the blood flow does not reach the brain, including ischemic stroke and hemorrhagic stroke, of which 87% are ischemic stroke (1). The results of the Global Burden of Disease Study (GBD) show that: In 2019, there were 3.94 million new cases of stroke in China, and the number of deaths due to stroke reached 2.19 million (2). In 2020, the mortality rate of stroke was 154.39/100,000, accounting for 22.8% of the total number of deaths (3). Stroke mortality in China is higher than in developed countries (such as the United States, the United Kingdom or Japan) (4), and is the third leading cause of death in most Western countries, while it is the first cause of death in China (5), and stroke is the sixth leading cause of death in the 25–49 age group (2). Studies have shown that intravenous thrombolysis within 3–4.5 h of the disease is the most effective treatment for Acute Ischemic Stroke (AIS) now (6, 7).

Middle-aged and young people are the key period of life development. People in this stage shoulder the heavy responsibility of family, which requires them to support the older adult and raise children, and realize their own life value (8). In recent years, the incidence of stroke in young and middle-aged people has continued to increase (9). If AIS occurs, symptoms such as hemiplegia, aphasia, and loss of consciousness may occur (10), which will be a huge blow to young and middle-aged people and their families. Pre-hospital delay is the earliest recorded time from the onset of symptoms to the arrival of the relevant department (11). In cases of frequent transient cerebral ischemia preceding a stroke, the onset of the last neurological injury is considered the starting point. In this study, based on relevant literature and thrombolysis timing, a stroke patient’s arrival at the hospital exceeding 3 h is regarded as a pre-hospital delay (12). Studies have shown that there is a high incidence of prehospital delay in AIS patients (13), and the delay in seeing a doctor is the main cause of AIS thrombosis (14). During the thrombolytic period, the prognosis of patients decreased by 10% every 15 min (15). Lansberg et al. (16) showed that within 3 h after the onset of AIS, the disability rate of AIS patients increased by 1% every 10 min. Therefore, how to reduce the patient’s medical treatment time has aroused extensive attention in the international community. Mellon et al. (17) conducted interviews with 149 consecutive patients with ischemic stroke through a questionnaire survey. The results indicated that respondents had limited knowledge about the symptoms and risk factors of ischemic stroke, which might be the reason for the failure to take immediate action when symptoms appeared, leading to delayed medical care. K Van et al. (18) explored the relationship between illness perception and coping styles and patient delay in seeking medical care. The results showed that passive coping styles were associated with patient delay in seeking medical care. Scholars abroad believe that patients with low levels of social support, when experiencing initial symptoms of stroke such as chest tightness, dizziness, limb numbness, etc., find it more difficult to obtain help from family, friends, colleagues, etc., which can easily lead to delayed medical care and serious consequences (12, 19).

Although many scholars have studied the factors related to the pre-hospital delay of AIS, most of them focus on the middle-aged and older adult people, and the trend of AIS becoming younger in recent years is obvious. At present, there is no relevant study on the pre-hospital delay of middle-aged and young AIS patients in China. Therefore, the objective of this study is to investigate the current situation of prehospital delay in young and middle-aged AIS patients in China, analyze the main influencing factors, and provide references for researchers to formulate intervention programs and implement intervention strategies to shorten prehospital delay in young and middle-aged AIS patients. To promote secondary prevention of AIS in young and middle-aged people.

## 2 Materials and methods

### 2.1 Study design and sample

A cluster random sampling method was used to select two hospitals from Grade III and Grade A hospitals in Hunan Province, and selected middle-aged and young AIS patients who met the criteria and volunteered to participate in this study from May 2023 to February 2024 from two Grade III hospitals as the study objects. Inclusion criteria: (1) AIS detected by CT or MRI; (2) According to the age classification of young and middle-aged people by WHO, that is, 18–59 years old; (3) Clear mind and stable state; Volunteer. Exclusion criteria: (1) AIS patients who had experienced mental or psychological illness prior to hospitalization; (2) AIS patients in critical condition, at constant risk of life-threatening situations; when combined with other severe diseases, such as heart failure, kidney failure, or respiratory failure.

### 2.2 Questionnaires

#### 2.2.1 General information questionnaire

After consulting the relevant literature, according to the purpose of the study, including the patient’s socio-demographic data and disease-related data. Including age, sex, ethnicity, marital status, education level, occupational status, residence, family per capita monthly income, medical payment method, location of onset, distance from onset to hospital, time of onset, first symptoms, mode of transport, whether referral, whether symptoms were first detected by others, etc.

#### 2.2.2 Social support rate scale (SSRS)

The scale, compiled by Xiao et al. (20), is widely utilized to assess the social support of research participants. The scale included 3 dimensions of objective support, subjective support and support utilization, with a total of 10 items. The scoring method is as follows: items 1 to 4, 8 to 10, single choice, each correct answer is counted as 1 to 4 points, and Article 5 is counted as the total score of items A, B, C, D and E5, each item from “none” to “fully supported” is counted as 1 to 4 points. Rules 6 and 7:0 points for answers to “no source,” and points for answers to “the following sources.” The total score of the scale is the sum of 10 items. The higher the score, the higher the social support degree. The reliability was 0.92, and Cronbach’s *α* coefficient was between 0.89 and 0.94.

#### 2.2.3 Health literacy scale for stroke patients

The scale was sinicized and revised by Liu et al. (21), and consisted of 3 dimensions (basic knowledge and concept, healthy lifestyle, and basic skills) with a total of 20 items. Likert 5-level scoring method was adopted for the scale (from “disagree” 1 point to “strongly agree with 5 points”). The total score of the scale was 100 points. The higher the score, the better the health literacy of the patients. The Cronbach’s *α* coefficient of the total table is 0.929, and the Cronbach’s α coefficient of each dimension is 0.926 ~ 0.942, which has good internal consistency.

#### 2.2.4 Medical coping modes questionnaire (MCMQ)

Developed by Professor Feifel et al. (22) in the United States, the questionnaire was Sinicized by Shen and Jiang (23) to evaluate the coping styles taken by patients in the face of diseases. In the face of coping styles, problem coping is emphasized, while avoidance and submission emphasize emotional coping. The scale consists of 3 dimensions and 20 items. Facing, avoiding and yielding were calculated separately. Each item is scored according to grades 1 to 4, among which Cronbach’s αcoefficients are 0.69, 0.60, and 0.76, respectively.

### 2.3 Data collection

Prior to the launch of this study, the investigators had received research support through full communication and coordination with the heads of relevant departments in the hospital. The investigators were clinical nurses with relevant professional knowledge or postgraduate students. The researchers trained them to briefly understand the purpose and content of the study, and standardized and unified the scoring method of the questionnaire. Questionnaires were collected by paper questionnaires. Before handing out the questionnaire, the researchers informed the subjects of the significance of the study for the purpose and the requirements for filling out the questionnaire. All respondents were surveyed with informed consent. The questionnaire takes about 20 min to complete. After the completion of the questionnaire, the investigators immediately collected the questionnaire, and verified and gave feedback on the content filled in the questionnaire. All investigations were conducted when the patient was in stable condition. After sorting out the collected questionnaires, if any omissions and errors are found, the respondents will be checked and corrected again within 24 h to ensure the authenticity and completeness of the questionnaires, and two copies will be entered into Excel 2019. In this study, a total of 700 questionnaires were distributed, and 671 were recovered, with an effective rate of 95.6%. The studies involving human participants were reviewed and approved by Biomedical Research Ethics Committee of Hunan Normal University, and the ethics number is 2023416. Written informed consent to participate in this study was provided by the patient.

### 2.4 Statistical analysis

SPSS26.0 software was used to process the data. All measurement data were tested for normality (Kolmogorov–Smirnov test) and homogeneity of variance (Levene test). The measurement data of normal distribution are expressed as x¯ ± s, and independent sample t test is used for inter-group comparison. Data with non-normal distribution were represented by M (Q1, Q3), and comparison between groups was performed by Wilcoxon rank sum test. The counting data were represented by cases or percentages, *χ*2 test was used for comparison between groups, and Fisher exact probability method was used when the expected frequency was less than 5. When analyzing the factors affecting the pre-hospital delay of AIS in young and middle-aged people, a single factor analysis was performed first. Variables with *p* < 0.05 were selected for binary stepwise logistic regression analysis, and the odds ratio (OR) of each factor and the corresponding 95%CI were calculated. *p* < 0.05 was considered to be statistically significant.

## 3 Results

### 3.1 General data comparison of AIS patients with prehospital delay group and non-delay group

A comparative analysis of the general demographic data of middle-aged and young AIS patients between the two groups showed that there were statistical differences between the two groups in terms of marital status, education, occupation, monthly income, physical examination, place of residence and living conditions (*p* < 0.05). See Table 1 for details. All data are categorical data and therefore chi-square tests are used. The theoretical frequency for Insurance is less than 5, thus Fisher’s exact test was performed.

**Table 1:** Compares the general demographic of young and middle-aged AIS patients (*n* = 671).

Variable		Non-delay group (*n* = 137)	Delay group (*n* = 534)	*χ^2^*	*p*
Age(years)	<45	51	188	0.194	0.660
	≥45	86	346		
Sex	Male	98	351	1.658	0.198
	Female	39	183		
Nation	The Han nationality	124	458	2.132	0.144
	Minority nationality	13	76		
Marital status	Unmarried	4	59	9.481	0.009
	Be married	103	386		
	Divorced or widowed	30	89		
Education	Below primary school	20	78	18.738	0.001
	Junior high school	10	99		
	High school/ Technical secondary school	39	143		
	Junior college	18	95		
	Bachelor degree or above	50	119		
Occupation	Retire	18	21	46.186	<0.001
	Peasant	9	129		
	Freelance work	43	222		
	Public institution	53	129		
	No occupation or other	14	33		
Monthly income (¥)	≤3,000	44	207	16.411	0.001
	3,001–6,000	37	200		
	6,001–9,000	21	50		
	>9,000	35	77		
Physical examination	Several times a year	4	16	8.650	0.034
	Once a year	83	269		
	Every few years	30	108		
	Never	20	141		
Place of residence	Village	24	188	22.389	<0.001
	City	52	204		
	Towns	61	142		
Residential status	Live with family	94	201	43.895	<0.001
	Live with friends or colleagues	30	192		
	Live alone	13	141		
Insurance	Medical insurance	136	518	2.268*	0.219
	Self-financing	1	16		

*shows fisher’s exact probability method.

### 3.2 The relationship between first symptoms and prehospital delay

Compared with the two groups, language disorder, dysphagia, vision disorder, headache and vomiting, consciousness disorder, limb numbness and weakness, the differences were statistically significant (*p* < 0.05). There was no significant difference in the symptoms of dizziness, walking instability, facial numbness and mouth angle skew between the two groups (*p* > 0.05). Relationship between initial symptoms and prehospital delay in two groups (see Table 2). All data are categorical data and therefore chi-square tests are used.

**Table 2:** Relationship between initial symptoms and prehospital delay (*n* = 671).

Variable	non-delay group (*n* = 137)	Delay group (*n* = 534)	*χ* ^2^	*p*
Language barrier	36	80	9.729	0.002
dysphagia	13	126	13.21	<0.001
Visual disturbance	15	138	13.74	<0.001
Dizziness or unsteady walking	48	199	0.233	0.629
Headache and vomiting	43	36	63.753	<0.001
Facial numbness	39	116	2.792	0.095
Disturbance of consciousness	88	106	104.497	<0.001
Limb numbness and weakness	59	329	31.128	<0.001

### 3.3 The relationship between disease-related factors and pre-hospital delay

Compared with other diseases related data of AIS patients in the pre-hospital delayed group and the non-delayed group, the results showed that there were statistical differences between the two groups in the location of onset, distance from the hospital for treatment, time of onset, mode of transport, whether to be referred, whether to be discovered by others, whether to receive training or learn stroke knowledge, whether to know stroke at the time of onset, whether to know that the earlier thrombolysis is better, and the cumulative number of underlying diseases (*p* < 0.05). For details, see Table 3. All data are categorical data and therefore chi-square tests are used.

**Table 3:** Relationship between other disease-related factors and prehospital delay (*n* = 671).

Variable		non-delay group (*n* = 137)	Delay group (*n* = 534)	χ^2^	*p*
Location of disease	Work unit	73	215	7.892	0.019
	At home	43	201		
	Public place	21	118		
Distance from onset to hospital	<10 km	52	48	78.274	<0.001
	10 ~ 20 km	64	288		
	>20 km	21	198		
Onset time	Daytime (6:00–18:00)	87	198	31.302	<0.001
	Evening (18:00–24:00)	36	252		
	Night (24:00–6:00)	14	84		
Transport mode	Ambulance transfer	59	139	15.263	<0.001
	Private car or taxi transfer	71	356		
	Public transport	7	39		
Referral or not	Yes	18	182	22.858	<0.001
	No	119	352		
Others have found	Yes	66	162	15.465	<0.001
	No	71	372		
Whether to receive training or learn about stroke	Received stroke related education	86	86	135.182	<0.001
	Have seen a stroke	46	265		
	Know nothing	5	183		
Whether it was known to be a stroke at the time of onset	Yes	93	65	187.971	<0.001
	No	44	469		
Know thrombolysis	Yes	17	214	72.858	<0.001
	No	16	120		
Cumulative number of underlying diseases	No previous medical conditions	17	214	72.858	<0.001
	Combine 1 species	16	120		
	Merge 2	47	117		
	Merge more than 3 kinds	57	83		

### 3.4 Comparison of social support, health literacy and coping styles between the delayed group and the non-delayed group of AIS patients before hospital

The study compared and analyzed the levels of social support among young and middle-aged patients with AIS across two groups, revealing statistically significant differences in the overall social support scores and scores across various dimensions between the groups (*p* < 0.05). A similar comparative analysis was conducted on the health literacy levels of middle-aged and young AIS patients, which also demonstrated statistically significant differences in the total health literacy scores and scores across various dimensions between the two groups (*p* < 0.05). Furthermore, an analysis of the coping styles of these patients indicated that there were significant statistical differences in terms of coping strategies, specifically in avoidance and yielding coping styles, between the two groups (*p* < 0.05), as illustrated in Table 4. After conducting normality tests and consulting with experts, it was determined that the scale scores were normally distributed or approximately normally distributed. Therefore, a t-test was used to measure the differences in scale scores between the delayed group and the non-delayed group.

**Table 4:** Comparison of social support, health literacy and coping styles between the two groups.

Variable	Non-delay group	Delay group	*t*	*p*
Objective support	10.5 ± 4.24	6.64 ± 2.63	10.477	<0.001
Subjective support	22.39 ± 4.37	16.52 ± 3.87	15.403	<0.001
Support availability	7.03 ± 2.33	4.98 ± 1.97	9.484	<0.001
Social support score	39.91 ± 8.8	28.14 ± 6.65	14.629	<0.001
Basic knowledge and philosophy	20.48 ± 3.58	15.63 ± 3.88	13.925	<0.001
Healthy lifestyle and behavior	25.97 ± 7.86	19.43 ± 5.26	9.221	<0.001
Basic skill	15.88 ± 5.35	9.82 ± 3.23	12.68	<0.001
Health literacy score	62.33 ± 12.02	44.87 ± 8.89	15.919	<0.001
Face	23.09 ± 2.96	20.14 ± 2.85	10.735	<0.001
Avoid	16.67 ± 3.43	18.02 ± 2.34	−4.332	<0.001
Yield	12.71 ± 2.03	13.73 ± 1.91	−5.499	<0.001

### 3.5 Multivariate analysis of pre-hospital delay in young and middle-aged AIS patients

The results are shown in Table 5. The variables included in the logistic regression equation were marital status, education level, occupation, monthly income (yuan), physical examination, place of residence, living conditions, location of onset, distance from the onset to the hospital, time of onset, mode of transport, whether to be referred, whether the symptoms were discovered by others, whether to receive training or learn stroke knowledge, whether to know it was stroke at the time of onset. Knowledge of whether earlier thrombolysis is better, number of underlying diseases, first symptoms (speech disorder, swallowing disorder, vision disorder, headache and vomiting, disturbance of consciousness, limb numbness and weakness), social support score, health literacy score, face coping style score, avoidance coping style score and submission coping style score. Among them, the factors that can extend the pre-hospital time are: The onset place was at home (OR = 4.997, *p* = 0.011, 95%CI: 1.444 ~ 17.285), the first symptoms were numbness and weakness of limbs (OR = 3.500, *p* = 0.027, 95%CI:1.155 ~ 10.605), avoidance coping style (OR = 1.298, *p* = 0.027, 95%CI:1.030 ~ 1.636) and submission coping style (OR = 1.721, *p* < 0.001, 95%CI: 1.269 ~ 2.333). The factors that can shorten the pre-hospital time of patients are: Stroke was known at the time of onset (OR = 0.120, *p* = 0.001, 95%CI:0.032 ~ 0.444), the first symptoms were headache and vomiting (OR = 0.052, *p* < 0.001, 95%CI:0.014 ~ 0.186), disturbance of consciousness (OR = 0.087, *p* < 0.001, 95%CI:0.024 ~ 0.322), and the level of social support (OR = 0.891, *p* = 0.001, 95%CI:0.830 ~ 0.956) and coping style (OR = 0.688, *p* < 0.001, 95%CI:0.570 ~ 0.831). See Table 5 for details.

**Table 5:** Multi-factor analysis of pre-hospital delay in AIS patients.

Variable	*B*	S.E	Wald	*p*	OR	95%CI
The place of onset was home	1.609	0.633	6.454	0.011	4.997	1.444 ~ 17.285
Stroke was known at the time of onset	−2.122	0.668	10.092	0.001	0.120	0.032 ~ 0.444
Headache and vomiting	−2.965	0.656	20.453	<0.001	0.052	0.014 ~ 0.186
Disturbance of consciousness	−2.442	0.668	13.374	<0.001	0.087	0.024 ~ 0.322
Limb numbness and weakness	1.253	0.566	4.904	0.027	3.500	1.155 ~ 10.605
Social support	−0.115	0.036	10.299	0.001	0.891	0.830 ~ 0.956
Health literacy	−0.066	0.025	6.969	0.008	0.936	0.891 ~ 0.983
Face	−0.374	0.096	15.147	<0.001	0.688	0.570 ~ 0.831
Avoid	0.261	0.118	4.887	0.027	1.298	1.030 ~ 1.636
Yield	0.543	0.155	12.222	<0.001	1.721	1.269 ~ 2.333
Constant	2.811	4.639	0.367	0.545	16.621	–

## 4 Discussion

The rate of pre-hospital delay among young and middle-aged patients suffering from ischemic stroke was alarmingly high at 79.6%. This issue is particularly concerning. This result is worse than that in other countries. For example, data from a multi-center study in the United States pointed out that only 21–40% of AIS patients can arrive at the hospital in time after the onset of stroke (24). Youth may be unaware of stroke symptoms, often mistaking it for an “older adult disease,” disregarding early indicators like sudden headaches, limb weakness, or speech difficulties. In Chinese culture, young individuals often endure discomfort, hoping symptoms will pass, causing delayed medical intervention. Additionally, some young patients rely on self-help strategies, like resting or medication, instead of seeking prompt professional medical assistance (25). Financial burden is also a consideration, especially in cases where medical insurance is unavailable or insufficient. Patients may choose to monitor the progression of their illness rather than seek immediate medical attention (26). Work stress or career concerns may delay medical attention for younger individuals. Mild or atypical symptoms in young stroke patients can be overlooked or misdiagnosed. Therefore, raising awareness of stroke symptoms among young people through media and community activities is crucial. Furthermore, optimizing primary medical services, enhancing the stroke identification and preliminary processing capabilities of grassroots institutions, simplifying procedures, establishing green channels for stroke patients to reduce delays, providing psychological support, strengthening social assistance systems, and improving medical insurance policies to alleviate the economic burdens of young patients are key measures to address this issue (27).

This study found that pre-hospital delay was more likely to occur at home than at work. This is consistent with the research results of Rivero et al. (28). Many may lack knowledge for stroke identification and emergency treatment at home, delaying emergency service activation. In work or public settings, more coworkers and witnesses often act swiftly by contacting services or providing assistance. This study found headache, vomiting, and consciousness disturbances catalyze timely medical attention seeking. Abrupt symptom onset and severity fluctuations correlate with reduced pre-hospital time. Studies show a protective relationship between condition severity and pre-hospital delay duration, attributed to accurate severity assessment leading to heightened vigilance and active medical assistance pursuit (29). Increased awareness can prompt earlier care, reducing potential delays. Mildly symptomatic patients often do not realize severity until symptoms worsen. Thus, universal education on initial stroke symptoms via various channels is necessary, enabling residents to identify and act timely, reducing pre-hospital delay due to neglect.

Health literacy is the basis for individuals to adopt correct health behaviors (30). In today’s society, with the acceleration of the pace of life and the increase in work pressure, the risk of stroke and other cardiovascular and cerebrovascular diseases among young and middle-aged people also increases (31–33). In this case, the popularization and dissemination of health knowledge is particularly important. Mastering certain health knowledge can not only help patients identify potential health problems, but also promote them to take the initiative to seek medical treatment, thereby winning valuable treatment opportunities. Zhao et al. (34) implemented the Stroke 1-2-0 stroke awareness program in China and found that after stroke awareness training, the public’s awareness of timely thrombolytic therapy was improved. When patients have a low level of health knowledge, they are also more likely to delay seeing a doctor (17, 35, 36). Our investigation further revealed that enhanced health literacy, encompassing a greater understanding of the disease, facilitated patients in seeking assistance more expeditiously. The reason is that health literacy encourages young and middle-aged people to seek professional medical assistance. With a certain level of health literacy, individuals can accurately assess their health and recognize the need for prompt medical attention. They understand stroke as an emergency and seek immediate medical help instead of waiting for symptoms to worsen. This proactive approach reduces delays in medical attention and maximizes the treatment window for stroke, improving treatment outcomes.

Social support is the emotional support an individual receives in a social network, as well as a subjective feeling of being respected, understood and sympathized with (37). Social support is an important factor affecting patients’ timely medical treatment. In this study, young and middle-aged stroke patients with lower social support were more likely to have pre-hospital delay. It is similar to the study of Yin et al. (38), their research suggests that a key part of delayed pre-hospital decisions may be a lack of internal motivation. The reason for this phenomenon is that high-quality social support can alleviate stress stimulation and reduce the damage caused by stress (39). Lower social support leads to greater sensitivity to environmental changes and reduced willingness to seek help. Under anticipation of or actual pressure, they are prone to display negative emotions and adopt negative coping strategies (40–42). Abundant social support offers emotional refuge and boosts psychological resilience. It equips individuals with more coping strategies and choices during pressure, fostering a positive problem-solving attitude and avoiding negative emotions, a concept widely recognized by the international academic community (39, 42, 43). Researchers suggest that patients with strong social support often have stable resources like financial aid and medical insurance, aiding timely access to medical care (38, 44). Family and friends’ encouragement is also key, speeding up treatment and reducing delays. Thus, a robust social support network is vital for stroke patients, enhancing their readiness to seek timely medical help and aiding faster recovery. A collaborative environment involving families, communities, and healthcare professionals can significantly improve patients’ quality of life, offering hope and confidence.

This study indicates that coping styles affect prehospital delay in middle-aged and young stroke patients, with active copers less likely to delay. Positive coping can help individuals manage stress, maintain and improve psychological and physical stability. Healthy living habits, a positive attitude, and professional psychological support can promote overall health (45). Illness, an inevitable part of life, can stress patients’ daily lives. This sudden shock threatens physical health and causes extreme panic and helplessness, prompting various coping strategies to reduce pain and anxiety. These may include self-soothing, seeking social support, changing the environment, or adjusting daily activities. However, negative coping styles can complicate the situation, further affecting the patient’s health. Van der Elst et al. (18) investigated the correlation between coping styles and pre-hospital delay, and found that there was a significant correlation between patients’ adverse coping behaviors and pre-hospital delay. This coincides with the conclusion of our study. Therefore, it is necessary to encourage young and middle-aged stroke patients to face their illness with a positive attitude, and face life with a smile, and face various challenges.

## 5 Conclusion

Factors contributing to these delays encompass the location of the stroke’s onset, the initial symptoms experienced, awareness of the stroke’s nature, the extent of social support, health literacy, and the coping strategies employed by the individuals. Consequently, it is imperative to concentrate on these determinants, enhance public education on stroke symptoms, and ensure timely identification of acute ischemic stroke (AIS). Moreover, collaborative support from various sectors is essential to elevate the health literacy and proactive coping mechanisms of young and middle-aged individuals affected by AIS.

It is also important to note that this study has its limitations. The participants were young and middle-aged AIS patients from two of China’s top three hospitals, which may not provide a fully representative sample. Future research should involve multi-center, large-scale studies across different regions and hospitals of varying levels to validate the findings. Given the constraints of time and resources, this study did not include an intervention component, but subsequent research should focus on controlled factors and incorporate intervention strategies.

## Data Availability

The raw data supporting the conclusions of this article will be made available by the authors, without undue reservation.
